# [3,3′-Bis(1-naphthylmethyl)-1,1′-(2,2′-oxy­diethyl­ene)bis­(imidazol-2-yl­idene)]mercury(II) bis­(hexa­fluorido­phosphate) acetonitrile solvate

**DOI:** 10.1107/S1600536809001743

**Published:** 2009-01-23

**Authors:** Wen-Yan Guo, Gui-Ying Dong

**Affiliations:** a Department of Chemistry, Shanxi Normal University, Linfen 041004, People’s Republic of China; b College of Chemical Engineering and Biotechnology, Hebei Polytechnic University, Tangshan 063009, People’s Republic of China

## Abstract

In the title compound, [Hg(C_32_H_30_N_4_O)](PF_6_)_2_·CH_3_CN, the mercury(II) ion is coordinated by two carbene C atoms [Hg—C = 2.060 (6) and 2.066 (6) Å] and one ether O atom [Hg—O = 2.561 (5) Å] in a distorted T-shaped geometry with a C—Hg—C angle of 166.3 (3)°. One hexa­fluorido­phosphate anion is rotationally disordered between two orientations with an approximate ratio of 2:1. The crystal packing exhibits weak inter­molecular C—H⋯F and C—H⋯N inter­actions.

## Related literature

For the crystal structures of related silver, gold and palladium complexes, see: Wang *et al.* (2005 ▶); Nielsen *et al.* (2006 ▶). For the details of synthesis of nucleophilic heterocyclic carbene ligands, see: Arduengo *et al.* (1991 ▶); Wang *et al.* (2006 ▶).
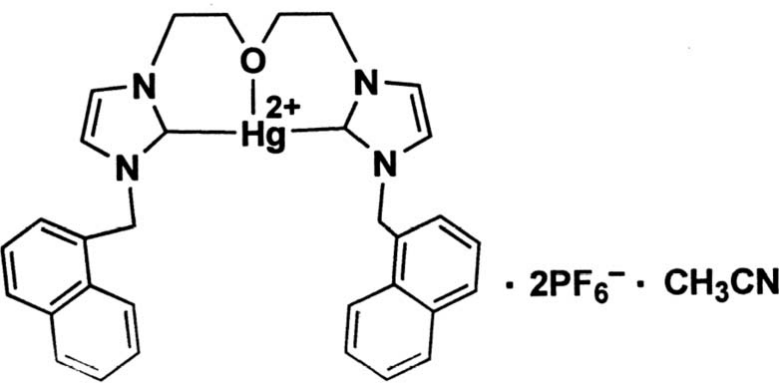


## Experimental

### 

#### Crystal data

[Hg(C_32_H_30_N4O)](PF_6_)_2_·C_2_H_3_N
*M* *_r_* = 1018.18
Monoclinic, 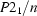

*a* = 9.204 (2) Å
*b* = 11.433 (3) Å
*c* = 35.922 (10) Å
β = 92.837 (5)°
*V* = 3775.7 (17) Å^3^
*Z* = 4
Mo *K*α radiation
μ = 4.26 mm^−1^
*T* = 293 (2) K
0.18 × 0.16 × 0.12 mm

#### Data collection

Bruker SMART CCD area-detector diffractometer
Absorption correction: multi-scan (*SADABS*; Sheldrick, 1996 ▶) *T*_min_ = 0.448, *T*_max_ = 0.590
21427 measured reflections
7729 independent reflections
5450 reflections with *I* > 2σ(*I*)
*R*_int_ = 0.062

#### Refinement

*R*[*F* ^2^ > 2σ(*F* ^2^)] = 0.054
*wR*(*F* ^2^) = 0.101
*S* = 1.12
7729 reflections
552 parameters
108 restraints
H-atom parameters constrained
Δρ_max_ = 0.82 e Å^−3^
Δρ_min_ = −1.46 e Å^−3^

### 

Data collection: *SMART* (Bruker, 1998 ▶); cell refinement: *SAINT* (Bruker, 1999 ▶); data reduction: *SAINT*; program(s) used to solve structure: *SHELXS97* (Sheldrick, 2008 ▶); program(s) used to refine structure: *SHELXL97* (Sheldrick, 2008 ▶); molecular graphics: *SHELXTL* (Sheldrick, 2008 ▶); software used to prepare material for publication: *SHELXTL*.

## Supplementary Material

Crystal structure: contains datablocks I, global. DOI: 10.1107/S1600536809001743/cv2505sup1.cif

Structure factors: contains datablocks I. DOI: 10.1107/S1600536809001743/cv2505Isup2.hkl

Additional supplementary materials:  crystallographic information; 3D view; checkCIF report

## Figures and Tables

**Table 1:** Hydrogen-bond geometry (Å, °)

*D*—H⋯*A*	*D*—H	H⋯*A*	*D*⋯*A*	*D*—H⋯*A*
C5—H5*A*⋯F3	0.97	2.50	3.384 (9)	151
C5—H5*B*⋯F7	0.97	2.39	3.307 (9)	157
C18—H18*A*⋯F4^i^	0.97	2.53	3.150 (10)	122
C22—H22*B*⋯F9^ii^	0.97	2.46	3.195 (11)	132
C24—H24⋯N4	0.93	2.50	2.847 (9)	103

Symmetry codes: (i) 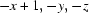; (ii) 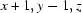.

**Fig. 1.**
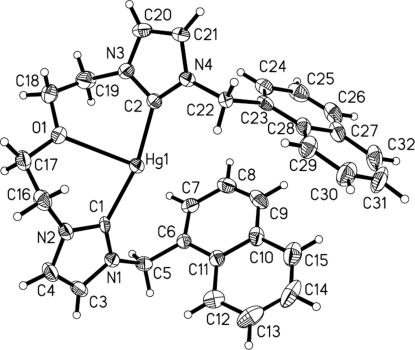
The molecular structure of the cation in (I) showing the atomic numbering and 30% probability displacement ellipsoids.

## supplementary crystallographic information

### Comment

Nucleophilic heterocyclic carbene (NHC) ligands have enjoyed wide applicability as ligands for transition metals in a variety of catalytic transformations since they were first isolated in 1991 (Arduengo *et al.*, 1991).The silver, gold and palladium complexes of bis-NHC ligands bearing a weakly coordinating ether functionality have been reported (Wang *et al.*, 2005; Nielsen *et al.*, 2006). To study further the coordination chemistry of this ligand, we report here the crystal structure of the title complex, (I).

The title compound, [Hg(C_32_H_30_N_4_O)](PF_6_)_2_[CH_3_CN] (I), crystallizes with one (*R*)-2,2'-bis[1*H*-imidazole-3-oxyethyl-1-(1-menaphthyl)]-1,1'-binaphthyl mercury (II) cation, two hexafluoridophosphate anions and one acetonitrile solvate molecule in an asymmetric unit. The cation of (I) is a 10-membered macrocyclic metal complex of naphthyl-carbene ligand adopting a *cis*-conformation. The geometry of the Hg(II) coordination is distorted T-shaped, formed by two C(carbene) atoms [Hg—C = 2.060 (6) and 2.066 (6) Å] and one ether oxygen atom with bond angle C2—Hg1—O1=83.6 (2)°, C1—Hg1—O1= 82.9 (2)° and C1—Hg1—C2=166.3 (3)°,respectively. The crystal packing exhibits weak intermolecular C—H···F and C—H···N interactions (Table 1).

### Experimental

The ligand 1,1'-(oxy-1,2-ethanediyl)bis[3-(1-naphthalenemethyl)imidazolium bis(hexafluoridophosphate) was prepared according to the reported procedure (Wang *et al.*, 2006). Anhydrous Hg(OAc)_2_ (29.5 mg, 0.10 mmol) was added to a solution of the corresponding diazolium salt (77.0 mg, 0.10 mmol) in acetonitrile (25 ml) under argon. The mixture was refluxed for 12 h and then cooled to the room temperature. the acetonitrile was removed *in vacuo* to give a white solid which was washed with methanol to give the crude product. White single crystals of the title compound were obtained by recrystallization from acetonitile and ethyl ether (yield: 90%) Anal. Calcd. for C_34_H_33_F_12_HgN_5_OP_2_: C, 40.11; H, 3.27; N, 6.88 Found: C, 40.10; H, 3.22; N 6.85

### Refinement

All H atoms were geometrically positioned [C—H= 0.93-0.96 Å] and refined as riding, with *U*_iso_(H) =1.2-1.5*U*_eq_(C). The six F atoms of one hexafluoridophosphate anion show rotational disorder, and they were refined as two groups sharing the same P atom with the occupancies refined to 0.676 (17) and 0.324 (17), respectively. The P—F and F—F  distances were restrained to 1.56 (1) and 2.21 (1) Å, respectively. The displacement parameters of the disordered F atoms were also restrained to be approximately isotropic.

### Crystal data

**Table:**

[Hg(C_32_H_30_N4O)](PF_6_)_2_·C_2_H_3_N	*F*(000) = 1992
*M**_r_* = 1018.18	*D*_x_ = 1.791 Mg m^−^^3^
Monoclinic, *P*2_1_/*n*	Mo *K*α radiation, λ = 0.71073 Å
*a* = 9.204 (2) Å	Cell parameters from 857 reflections
*b* = 11.433 (3) Å	θ = 2.3–22.3°
*c* = 35.922 (10) Å	µ = 4.26 mm^−^^1^
β = 92.837 (5)°	*T* = 293 K
*V* = 3775.7 (17) Å^3^	Block, white
*Z* = 4	0.18 × 0.16 × 0.12 mm

### Data collection

**Table:**

Bruker SMART CCD area-detector diffractometer	7729 independent reflections
Radiation source: fine-focus sealed tube	5450 reflections with *I* > 2σ(*I*)
graphite	*R*_int_ = 0.062
φ and ω scans	θ_max_ = 26.4°, θ_min_ = 2.3°
Absorption correction: multi-scan (*SADABS*; Sheldrick, 1996)	*h* = −11→11
*T*_min_ = 0.448, *T*_max_ = 0.590	*k* = −14→13
21427 measured reflections	*l* = −44→28

### Refinement

**Table:**

Refinement on *F*^2^	Primary atom site location: structure-invariant direct methods
Least-squares matrix: full	Secondary atom site location: difference Fourier map
*R*[*F*^2^ > 2σ(*F*^2^)] = 0.054	Hydrogen site location: inferred from neighbouring sites
*wR*(*F*^2^) = 0.101	H-atom parameters constrained
*S* = 1.12	*w* = 1/[σ^2^(*F*_o_^2^) + (0.0346*P*)^2^ + 0.5948*P*] where *P* = (*F*_o_^2^ + 2*F*_c_^2^)/3
7729 reflections	(Δ/σ)_max_ = 0.001
552 parameters	Δρ_max_ = 0.82 e Å^−^^3^
108 restraints	Δρ_min_ = −1.46 e Å^−^^3^

### Special details

**Table:**

Geometry. All e.s.d.'s (except the e.s.d. in the dihedral angle between two l.s. planes) are estimated using the full covariance matrix. The cell e.s.d.'s are taken into account individually in the estimation of e.s.d.'s in distances, angles and torsion angles; correlations between e.s.d.'s in cell parameters are only used when they are defined by crystal symmetry. An approximate (isotropic) treatment of cell e.s.d.'s is used for estimating e.s.d.'s involving l.s. planes.
Refinement. Refinement of *F*^2^ against ALL reflections. The weighted *R*-factor *wR* and goodness of fit *S* are based on *F*^2^, conventional *R*-factors *R* are based on *F*, with *F* set to zero for negative *F*^2^. The threshold expression of *F*^2^ > σ(*F*^2^) is used only for calculating *R*-factors(gt) *etc*. and is not relevant to the choice of reflections for refinement. *R*-factors based on *F*^2^ are statistically about twice as large as those based on *F*, and *R*- factors based on ALL data will be even larger.

### Fractional atomic coordinates and isotropic or equivalent isotropic displacement parameters (Å^2^)

**Table:**

	*x*	*y*	*z*	*U*_iso_*/*U*_eq_	Occ. (<1)
Hg1	0.78607 (3)	0.22825 (2)	0.087425 (8)	0.04048 (10)	
N1	0.6181 (6)	0.4642 (5)	0.09040 (16)	0.0405 (14)	
N2	0.7257 (6)	0.4380 (5)	0.03912 (17)	0.0446 (14)	
N3	0.8152 (6)	−0.0190 (5)	0.06247 (17)	0.0456 (15)	
N4	0.9815 (6)	0.0114 (4)	0.10573 (16)	0.0400 (14)	
O1	0.7511 (5)	0.1912 (4)	0.01725 (14)	0.0562 (14)	
C1	0.7067 (7)	0.3919 (6)	0.07289 (18)	0.0356 (15)	
C2	0.8659 (7)	0.0599 (5)	0.08832 (19)	0.0364 (16)	
C3	0.5818 (8)	0.5568 (6)	0.0675 (2)	0.053 (2)	
H3	0.5218	0.6193	0.0730	0.063*	
C4	0.6487 (8)	0.5400 (6)	0.0362 (2)	0.055 (2)	
H4	0.6439	0.5894	0.0156	0.066*	
C5	0.5474 (7)	0.4420 (6)	0.1255 (2)	0.0472 (18)	
H5A	0.4552	0.4033	0.1198	0.057*	
H5B	0.5264	0.5167	0.1369	0.057*	
C6	0.6331 (7)	0.3695 (6)	0.15335 (19)	0.0401 (16)	
C7	0.5962 (8)	0.2543 (6)	0.1577 (2)	0.0507 (19)	
H7	0.5226	0.2219	0.1423	0.061*	
C8	0.6666 (9)	0.1856 (7)	0.1845 (2)	0.059 (2)	
H8	0.6406	0.1074	0.1868	0.071*	
C9	0.7719 (9)	0.2303 (8)	0.2072 (2)	0.064 (2)	
H9	0.8177	0.1824	0.2251	0.077*	
C10	0.8150 (7)	0.3472 (7)	0.2046 (2)	0.051 (2)	
C11	0.7459 (7)	0.4197 (6)	0.1767 (2)	0.0437 (17)	
C12	0.7841 (8)	0.5398 (7)	0.1752 (3)	0.062 (2)	
H12	0.7393	0.5886	0.1574	0.075*	
C13	0.8879 (10)	0.5835 (9)	0.2004 (3)	0.083 (3)	
H13	0.9133	0.6621	0.1993	0.100*	
C14	0.9563 (11)	0.5114 (11)	0.2276 (3)	0.093 (4)	
H14	1.0252	0.5429	0.2446	0.111*	
C15	0.9225 (9)	0.3971 (10)	0.2293 (3)	0.077 (3)	
H15	0.9707	0.3499	0.2471	0.093*	
C16	0.8153 (8)	0.3898 (7)	0.0104 (2)	0.056 (2)	
H16A	0.9099	0.3698	0.0218	0.068*	
H16B	0.8301	0.4498	−0.0081	0.068*	
C17	0.7529 (10)	0.2839 (7)	−0.0088 (2)	0.065 (2)	
H17A	0.6549	0.3001	−0.0186	0.077*	
H17B	0.8116	0.2625	−0.0294	0.077*	
C18	0.7271 (9)	0.0771 (7)	0.0039 (2)	0.063 (2)	
H18A	0.8134	0.0488	−0.0076	0.076*	
H18B	0.6473	0.0771	−0.0148	0.076*	
C19	0.6917 (8)	−0.0015 (7)	0.0353 (3)	0.066 (3)	
H19A	0.6106	0.0314	0.0480	0.080*	
H19B	0.6612	−0.0769	0.0252	0.080*	
C20	0.8994 (8)	−0.1176 (6)	0.0652 (2)	0.058 (2)	
H20	0.8875	−0.1850	0.0509	0.069*	
C21	1.0027 (8)	−0.0991 (6)	0.0926 (2)	0.052 (2)	
H21	1.0745	−0.1515	0.1009	0.062*	
C22	1.0724 (7)	0.0685 (5)	0.13471 (18)	0.0384 (16)	
H22A	1.0608	0.1525	0.1320	0.046*	
H22B	1.1733	0.0502	0.1307	0.046*	
C23	1.0407 (7)	0.0353 (6)	0.1741 (2)	0.0405 (17)	
C24	0.9391 (8)	−0.0466 (6)	0.1815 (2)	0.052 (2)	
H24	0.8897	−0.0861	0.1621	0.063*	
C25	0.9092 (10)	−0.0711 (7)	0.2189 (3)	0.068 (3)	
H25	0.8407	−0.1281	0.2238	0.082*	
C26	0.9761 (10)	−0.0150 (8)	0.2475 (3)	0.072 (3)	
H26	0.9535	−0.0326	0.2718	0.087*	
C27	1.0806 (9)	0.0706 (8)	0.2406 (2)	0.060 (2)	
C28	1.1169 (7)	0.0954 (6)	0.2036 (2)	0.0460 (18)	
C29	1.2233 (8)	0.1818 (7)	0.1976 (2)	0.060 (2)	
H29	1.2519	0.1968	0.1736	0.072*	
C30	1.2843 (9)	0.2434 (8)	0.2272 (3)	0.079 (3)	
H30	1.3532	0.3006	0.2229	0.095*	
C31	1.2465 (12)	0.2229 (11)	0.2629 (3)	0.099 (4)	
H31	1.2874	0.2675	0.2824	0.118*	
C32	1.1476 (11)	0.1357 (10)	0.2701 (3)	0.086 (3)	
H32	1.1251	0.1199	0.2946	0.103*	
P1	0.2485 (2)	0.25046 (17)	0.05306 (6)	0.0565 (6)	
F1	0.0984 (6)	0.2867 (5)	0.07142 (18)	0.1065 (19)	
F2	0.2972 (6)	0.3825 (4)	0.05426 (15)	0.0916 (17)	
F3	0.3110 (7)	0.2339 (5)	0.09374 (17)	0.121 (2)	
F4	0.1948 (7)	0.1215 (4)	0.05251 (18)	0.121 (2)	
F5	0.1711 (8)	0.2736 (6)	0.01355 (16)	0.126 (2)	
F6	0.3852 (7)	0.2175 (6)	0.0336 (3)	0.168 (4)	
P2	0.4756 (3)	0.85219 (19)	0.12903 (7)	0.0684 (7)	
F7	0.4630 (11)	0.7192 (5)	0.1395 (2)	0.098 (4)	0.676 (17)
F8	0.5350 (14)	0.8758 (9)	0.1705 (2)	0.142 (6)	0.676 (17)
F9	0.3183 (8)	0.8715 (10)	0.1421 (4)	0.160 (7)	0.676 (17)
F10	0.4176 (16)	0.8252 (9)	0.0891 (2)	0.162 (7)	0.676 (17)
F11	0.6340 (8)	0.8292 (11)	0.1181 (4)	0.167 (7)	0.676 (17)
F12	0.4887 (16)	0.9829 (5)	0.1198 (3)	0.170 (7)	0.676 (17)
F7'	0.580 (2)	0.779 (2)	0.1544 (7)	0.25 (2)	0.324 (17)
F8'	0.390 (3)	0.8943 (16)	0.1622 (5)	0.136 (12)	0.324 (17)
F9'	0.372 (2)	0.7449 (17)	0.1231 (6)	0.213 (19)	0.324 (17)
F10'	0.558 (2)	0.8116 (14)	0.0936 (5)	0.118 (11)	0.324 (17)
F11'	0.579 (2)	0.9594 (16)	0.1324 (6)	0.137 (12)	0.324 (17)
F12'	0.370 (2)	0.925 (2)	0.1017 (6)	0.154 (13)	0.324 (17)
N5	−0.0008 (12)	0.5965 (9)	0.0750 (3)	0.119 (4)	
C33	0.0674 (11)	0.5635 (10)	0.1002 (4)	0.093 (4)	
C34	0.1418 (12)	0.5175 (13)	0.1326 (4)	0.151 (6)	
H34A	0.1599	0.5791	0.1503	0.226*	
H34B	0.2326	0.4841	0.1259	0.226*	
H34C	0.0831	0.4582	0.1433	0.226*	

### Atomic displacement parameters (Å^2^)

**Table:**

	*U*^11^	*U*^22^	*U*^33^	*U*^12^	*U*^13^	*U*^23^
Hg1	0.04380 (15)	0.03932 (15)	0.03823 (15)	0.00852 (13)	0.00125 (10)	0.00183 (15)
N1	0.042 (3)	0.040 (3)	0.040 (4)	0.007 (3)	0.000 (3)	0.001 (3)
N2	0.053 (4)	0.043 (3)	0.038 (4)	0.002 (3)	0.002 (3)	0.010 (3)
N3	0.047 (3)	0.045 (4)	0.044 (4)	0.002 (3)	−0.001 (3)	−0.006 (3)
N4	0.039 (3)	0.036 (3)	0.045 (4)	0.004 (2)	0.000 (3)	0.002 (3)
O1	0.080 (4)	0.049 (3)	0.038 (3)	0.005 (3)	−0.003 (3)	−0.004 (2)
C1	0.038 (4)	0.045 (4)	0.023 (4)	0.004 (3)	−0.006 (3)	0.006 (3)
C2	0.036 (4)	0.035 (4)	0.038 (4)	−0.001 (3)	0.003 (3)	0.004 (3)
C3	0.064 (5)	0.038 (4)	0.055 (5)	0.011 (3)	0.004 (4)	0.012 (4)
C4	0.067 (5)	0.052 (5)	0.047 (5)	0.009 (4)	0.004 (4)	0.020 (4)
C5	0.045 (4)	0.045 (4)	0.052 (5)	0.015 (3)	0.007 (4)	−0.005 (4)
C6	0.040 (4)	0.047 (4)	0.034 (4)	0.013 (3)	0.006 (3)	−0.003 (3)
C7	0.058 (4)	0.043 (5)	0.051 (5)	−0.002 (3)	0.012 (4)	−0.007 (4)
C8	0.071 (5)	0.043 (4)	0.065 (6)	0.005 (4)	0.013 (5)	0.006 (4)
C9	0.062 (5)	0.069 (6)	0.062 (6)	0.029 (5)	0.015 (4)	0.019 (5)
C10	0.034 (4)	0.076 (6)	0.043 (5)	0.015 (4)	0.001 (3)	−0.004 (4)
C11	0.045 (4)	0.053 (5)	0.034 (4)	0.010 (3)	0.010 (3)	−0.002 (4)
C12	0.061 (5)	0.055 (5)	0.071 (6)	−0.003 (4)	0.009 (5)	−0.006 (5)
C13	0.070 (6)	0.085 (7)	0.096 (9)	−0.028 (5)	0.020 (6)	−0.039 (6)
C14	0.073 (7)	0.138 (10)	0.065 (8)	−0.012 (7)	−0.010 (6)	−0.047 (7)
C15	0.059 (6)	0.112 (8)	0.060 (6)	0.017 (5)	−0.006 (5)	−0.019 (6)
C16	0.061 (5)	0.058 (5)	0.051 (5)	0.002 (4)	0.014 (4)	0.018 (4)
C17	0.085 (6)	0.070 (6)	0.039 (5)	0.025 (5)	0.006 (4)	0.010 (5)
C18	0.072 (6)	0.063 (6)	0.053 (6)	0.004 (4)	−0.020 (5)	−0.009 (5)
C19	0.057 (5)	0.057 (5)	0.083 (7)	−0.001 (4)	−0.021 (5)	−0.016 (5)
C20	0.069 (5)	0.039 (4)	0.065 (6)	0.009 (4)	0.000 (5)	−0.013 (4)
C21	0.060 (5)	0.038 (4)	0.057 (5)	0.015 (3)	−0.003 (4)	−0.004 (4)
C22	0.039 (4)	0.040 (4)	0.036 (4)	0.003 (3)	−0.002 (3)	0.002 (3)
C23	0.045 (4)	0.036 (4)	0.042 (4)	0.011 (3)	0.011 (3)	0.014 (3)
C24	0.054 (5)	0.047 (4)	0.056 (5)	0.012 (4)	0.011 (4)	0.010 (4)
C25	0.075 (6)	0.061 (6)	0.070 (7)	0.013 (4)	0.025 (5)	0.024 (5)
C26	0.083 (7)	0.096 (7)	0.041 (6)	0.033 (6)	0.024 (5)	0.031 (5)
C27	0.057 (5)	0.090 (6)	0.035 (5)	0.035 (5)	0.011 (4)	0.010 (5)
C28	0.037 (4)	0.062 (5)	0.039 (5)	0.016 (3)	0.001 (3)	0.001 (4)
C29	0.046 (4)	0.081 (6)	0.052 (5)	−0.001 (4)	0.001 (4)	−0.008 (5)
C30	0.061 (5)	0.101 (7)	0.074 (7)	0.006 (5)	−0.015 (5)	−0.031 (6)
C31	0.086 (7)	0.149 (11)	0.058 (7)	0.038 (7)	−0.028 (6)	−0.054 (7)
C32	0.081 (7)	0.142 (10)	0.034 (5)	0.047 (7)	0.001 (5)	−0.001 (6)
P1	0.0708 (13)	0.0512 (15)	0.0477 (12)	−0.0113 (10)	0.0043 (10)	0.0032 (10)
F1	0.093 (4)	0.110 (4)	0.119 (5)	0.002 (3)	0.026 (4)	0.010 (4)
F2	0.134 (5)	0.059 (3)	0.081 (4)	−0.030 (3)	−0.003 (3)	0.009 (3)
F3	0.151 (6)	0.117 (5)	0.088 (5)	−0.046 (4)	−0.053 (4)	0.043 (4)
F4	0.188 (6)	0.060 (3)	0.120 (5)	−0.041 (4)	0.051 (5)	−0.020 (3)
F5	0.150 (6)	0.161 (6)	0.066 (4)	−0.056 (4)	−0.020 (4)	0.025 (4)
F6	0.110 (5)	0.127 (5)	0.277 (11)	0.016 (4)	0.108 (6)	0.001 (6)
P2	0.0722 (15)	0.0515 (15)	0.0814 (19)	0.0090 (11)	0.0026 (14)	−0.0021 (13)
F7	0.128 (9)	0.046 (5)	0.121 (9)	0.015 (5)	0.034 (7)	−0.006 (5)
F8	0.202 (15)	0.134 (10)	0.085 (8)	−0.020 (9)	−0.058 (9)	−0.010 (7)
F9	0.091 (8)	0.187 (13)	0.202 (17)	0.079 (8)	−0.004 (9)	−0.024 (12)
F10	0.267 (19)	0.130 (10)	0.080 (8)	0.018 (12)	−0.080 (10)	−0.015 (7)
F11	0.083 (8)	0.218 (16)	0.204 (17)	−0.027 (8)	0.053 (9)	0.003 (13)
F12	0.32 (2)	0.051 (6)	0.136 (11)	−0.031 (8)	−0.061 (12)	0.013 (6)
F7'	0.21 (3)	0.21 (3)	0.32 (4)	0.08 (3)	−0.04 (3)	0.11 (3)
F8'	0.15 (2)	0.16 (2)	0.10 (2)	0.067 (19)	0.042 (18)	−0.009 (16)
F9'	0.16 (3)	0.18 (3)	0.31 (4)	−0.11 (2)	0.05 (3)	0.01 (3)
F10'	0.113 (18)	0.072 (13)	0.18 (3)	−0.033 (13)	0.078 (17)	−0.066 (15)
F11'	0.14 (2)	0.16 (2)	0.11 (2)	−0.084 (18)	−0.024 (16)	−0.043 (17)
F12'	0.128 (19)	0.19 (3)	0.14 (2)	0.061 (19)	−0.023 (17)	0.02 (2)
N5	0.136 (9)	0.095 (7)	0.127 (10)	0.007 (7)	0.004 (8)	−0.008 (7)
C33	0.060 (7)	0.101 (9)	0.117 (11)	0.010 (6)	−0.003 (7)	−0.043 (8)
C34	0.081 (8)	0.253 (17)	0.116 (12)	0.056 (10)	−0.013 (8)	−0.055 (12)

### Geometric parameters (Å, °)

**Table:**

Hg1—C2	2.060 (6)	C19—H19A	0.9700
Hg1—C1	2.066 (6)	C19—H19B	0.9700
Hg1—O1	2.561 (5)	C20—C21	1.349 (10)
N1—C1	1.340 (8)	C20—H20	0.9300
N1—C3	1.371 (8)	C21—H21	0.9300
N1—C5	1.471 (9)	C22—C23	1.508 (9)
N2—C1	1.342 (8)	C22—H22A	0.9700
N2—C4	1.366 (8)	C22—H22B	0.9700
N2—C16	1.459 (9)	C23—C24	1.358 (9)
N3—C2	1.360 (8)	C23—C28	1.419 (10)
N3—C20	1.369 (8)	C24—C25	1.413 (11)
N3—C19	1.476 (9)	C24—H24	0.9300
N4—C2	1.328 (8)	C25—C26	1.335 (12)
N4—C21	1.366 (8)	C25—H25	0.9300
N4—C22	1.458 (8)	C26—C27	1.403 (12)
O1—C18	1.403 (8)	C26—H26	0.9300
O1—C17	1.414 (8)	C27—C32	1.412 (12)
C3—C4	1.324 (10)	C27—C28	1.416 (10)
C3—H3	0.9300	C28—C29	1.415 (10)
C4—H4	0.9300	C29—C30	1.374 (11)
C5—C6	1.494 (9)	C29—H29	0.9300
C5—H5A	0.9700	C30—C31	1.365 (14)
C5—H5B	0.9700	C30—H30	0.9300
C6—C7	1.370 (9)	C31—C32	1.383 (14)
C6—C11	1.423 (9)	C31—H31	0.9300
C7—C8	1.379 (10)	C32—H32	0.9300
C7—H7	0.9300	P1—F6	1.516 (6)
C8—C9	1.336 (11)	P1—F4	1.555 (5)
C8—H8	0.9300	P1—F3	1.555 (6)
C9—C10	1.399 (11)	P1—F2	1.575 (5)
C9—H9	0.9300	P1—F5	1.579 (6)
C10—C15	1.416 (11)	P1—F1	1.614 (6)
C10—C11	1.427 (10)	P2—F8'	1.536 (8)
C11—C12	1.419 (10)	P2—F12	1.537 (6)
C12—C13	1.377 (11)	P2—F10	1.538 (6)
C12—H12	0.9300	P2—F7'	1.539 (8)
C13—C14	1.405 (14)	P2—F11	1.551 (6)
C13—H13	0.9300	P2—F11'	1.555 (8)
C14—C15	1.346 (13)	P2—F9	1.560 (7)
C14—H14	0.9300	P2—F9'	1.561 (8)
C15—H15	0.9300	P2—F7	1.572 (6)
C16—C17	1.495 (10)	P2—F10'	1.582 (8)
C16—H16A	0.9700	P2—F12'	1.585 (8)
C16—H16B	0.9700	P2—F8	1.585 (6)
C17—H17A	0.9700	N5—C33	1.141 (13)
C17—H17B	0.9700	C33—C34	1.421 (16)
C18—C19	1.489 (11)	C34—H34A	0.9600
C18—H18A	0.9700	C34—H34B	0.9600
C18—H18B	0.9700	C34—H34C	0.9600
			
C2—Hg1—C1	166.3 (3)	C24—C25—H25	118.9
C2—Hg1—O1	83.6 (2)	C25—C26—C27	119.5 (8)
C1—Hg1—O1	82.9 (2)	C25—C26—H26	120.2
C1—N1—C3	109.3 (6)	C27—C26—H26	120.2
C1—N1—C5	127.0 (5)	C26—C27—C32	120.9 (9)
C3—N1—C5	122.9 (6)	C26—C27—C28	120.1 (8)
C1—N2—C4	108.4 (6)	C32—C27—C28	119.0 (9)
C1—N2—C16	126.4 (6)	C29—C28—C27	118.8 (8)
C4—N2—C16	125.2 (6)	C29—C28—C23	123.0 (7)
C2—N3—C20	108.8 (6)	C27—C28—C23	118.3 (7)
C2—N3—C19	126.1 (6)	C30—C29—C28	119.9 (9)
C20—N3—C19	125.1 (6)	C30—C29—H29	120.1
C2—N4—C21	110.4 (6)	C28—C29—H29	120.1
C2—N4—C22	124.6 (5)	C31—C30—C29	121.8 (10)
C21—N4—C22	125.1 (6)	C31—C30—H30	119.1
C18—O1—C17	118.5 (6)	C29—C30—H30	119.1
C18—O1—Hg1	120.0 (4)	C30—C31—C32	120.0 (9)
C17—O1—Hg1	121.5 (4)	C30—C31—H31	120.0
N1—C1—N2	106.9 (5)	C32—C31—H31	120.0
N1—C1—Hg1	131.0 (5)	C31—C32—C27	120.5 (9)
N2—C1—Hg1	121.6 (5)	C31—C32—H32	119.8
N4—C2—N3	106.6 (5)	C27—C32—H32	119.8
N4—C2—Hg1	132.4 (5)	F6—P1—F4	91.7 (4)
N3—C2—Hg1	119.9 (5)	F6—P1—F3	97.2 (5)
C4—C3—N1	106.8 (6)	F4—P1—F3	90.0 (3)
C4—C3—H3	126.6	F6—P1—F2	90.5 (3)
N1—C3—H3	126.6	F4—P1—F2	177.8 (4)
C3—C4—N2	108.6 (7)	F3—P1—F2	89.9 (3)
C3—C4—H4	125.7	F6—P1—F5	88.6 (5)
N2—C4—H4	125.7	F4—P1—F5	91.1 (3)
N1—C5—C6	115.4 (5)	F3—P1—F5	174.1 (4)
N1—C5—H5A	108.4	F2—P1—F5	88.8 (3)
C6—C5—H5A	108.4	F6—P1—F1	176.7 (5)
N1—C5—H5B	108.4	F4—P1—F1	88.3 (3)
C6—C5—H5B	108.4	F3—P1—F1	86.1 (4)
H5A—C5—H5B	107.5	F2—P1—F1	89.5 (3)
C7—C6—C11	119.9 (6)	F5—P1—F1	88.1 (4)
C7—C6—C5	119.0 (7)	F8'—P2—F12	84.9 (9)
C11—C6—C5	121.0 (6)	F8'—P2—F10	128.3 (8)
C6—C7—C8	121.1 (7)	F12—P2—F10	91.2 (4)
C6—C7—H7	119.5	F8'—P2—F7'	92.3 (6)
C8—C7—H7	119.5	F12—P2—F7'	127.2 (9)
C9—C8—C7	120.6 (7)	F10—P2—F7'	128.3 (10)
C9—C8—H8	119.7	F8'—P2—F11	140.5 (8)
C7—C8—H8	119.7	F12—P2—F11	91.5 (5)
C8—C9—C10	121.7 (7)	F10—P2—F11	91.0 (5)
C8—C9—H9	119.2	F7'—P2—F11	59.3 (9)
C10—C9—H9	119.2	F8'—P2—F11'	91.6 (6)
C9—C10—C15	122.4 (8)	F12—P2—F11'	36.5 (8)
C9—C10—C11	118.9 (7)	F10—P2—F11'	114.5 (8)
C15—C10—C11	118.7 (8)	F7'—P2—F11'	91.2 (6)
C12—C11—C6	122.9 (7)	F11—P2—F11'	64.6 (8)
C12—C11—C10	119.1 (7)	F8'—P2—F9	37.4 (8)
C6—C11—C10	117.8 (7)	F12—P2—F9	90.6 (5)
C13—C12—C11	119.4 (8)	F10—P2—F9	91.4 (4)
C13—C12—H12	120.3	F7'—P2—F9	117.5 (9)
C11—C12—H12	120.3	F11—P2—F9	176.7 (5)
C12—C13—C14	121.2 (9)	F11'—P2—F9	116.1 (8)
C12—C13—H13	119.4	F8'—P2—F9'	91.2 (6)
C14—C13—H13	119.4	F12—P2—F9'	142.0 (9)
C15—C14—C13	120.3 (9)	F10—P2—F9'	62.3 (9)
C15—C14—H14	119.8	F7'—P2—F9'	90.6 (6)
C13—C14—H14	119.8	F11—P2—F9'	114.1 (8)
C14—C15—C10	121.2 (9)	F11'—P2—F9'	176.7 (7)
C14—C15—H15	119.4	F9—P2—F9'	65.3 (8)
C10—C15—H15	119.4	F8'—P2—F7	94.1 (8)
N2—C16—C17	114.5 (6)	F12—P2—F7	178.6 (5)
N2—C16—H16A	108.6	F10—P2—F7	90.2 (4)
C17—C16—H16A	108.6	F7'—P2—F7	51.9 (10)
N2—C16—H16B	108.6	F11—P2—F7	88.7 (4)
C17—C16—H16B	108.6	F11'—P2—F7	142.7 (9)
H16A—C16—H16B	107.6	F9—P2—F7	89.1 (4)
O1—C17—C16	108.5 (6)	F9'—P2—F7	38.8 (9)
O1—C17—H17A	110.0	F8'—P2—F10'	177.4 (7)
C16—C17—H17A	110.0	F12—P2—F10'	93.8 (8)
O1—C17—H17B	110.0	F10—P2—F10'	49.4 (7)
C16—C17—H17B	110.0	F7'—P2—F10'	90.3 (5)
H17A—C17—H17B	108.4	F11—P2—F10'	41.7 (7)
O1—C18—C19	109.8 (7)	F11'—P2—F10'	88.7 (5)
O1—C18—H18A	109.7	F9—P2—F10'	140.6 (8)
C19—C18—H18A	109.7	F9'—P2—F10'	88.5 (5)
O1—C18—H18B	109.7	F7—P2—F10'	87.2 (7)
C19—C18—H18B	109.7	F8'—P2—F12'	89.8 (5)
H18A—C18—H18B	108.2	F12—P2—F12'	53.5 (8)
N3—C19—C18	113.1 (6)	F10—P2—F12'	49.7 (8)
N3—C19—H19A	109.0	F7'—P2—F12'	177.9 (7)
C18—C19—H19A	109.0	F11—P2—F12'	119.2 (8)
N3—C19—H19B	109.0	F11'—P2—F12'	89.3 (5)
C18—C19—H19B	109.0	F9—P2—F12'	64.1 (8)
H19A—C19—H19B	107.8	F9'—P2—F12'	88.8 (5)
C21—C20—N3	107.4 (6)	F7—P2—F12'	127.5 (9)
C21—C20—H20	126.3	F10'—P2—F12'	87.7 (5)
N3—C20—H20	126.3	F8'—P2—F8	51.9 (8)
C20—C21—N4	106.8 (6)	F12—P2—F8	90.6 (4)
C20—C21—H21	126.6	F10—P2—F8	178.2 (5)
N4—C21—H21	126.6	F7'—P2—F8	50.3 (10)
N4—C22—C23	115.2 (5)	F11—P2—F8	88.9 (4)
N4—C22—H22A	108.5	F11'—P2—F8	67.0 (8)
C23—C22—H22A	108.5	F9—P2—F8	88.6 (4)
N4—C22—H22B	108.5	F9'—P2—F8	116.2 (9)
C23—C22—H22B	108.5	F7—P2—F8	88.1 (4)
H22A—C22—H22B	107.5	F10'—P2—F8	130.4 (8)
C24—C23—C28	120.5 (7)	F12'—P2—F8	131.7 (9)
C24—C23—C22	121.6 (7)	N5—C33—C34	175.2 (15)
C28—C23—C22	117.8 (6)	C33—C34—H34A	109.5
C23—C24—C25	119.4 (8)	C33—C34—H34B	109.5
C23—C24—H24	120.3	H34A—C34—H34B	109.5
C25—C24—H24	120.3	C33—C34—H34C	109.5
C26—C25—C24	122.2 (8)	H34A—C34—H34C	109.5
C26—C25—H25	118.9	H34B—C34—H34C	109.5
			
C2—Hg1—O1—C18	28.4 (5)	C9—C10—C11—C6	1.7 (10)
C1—Hg1—O1—C18	−153.7 (5)	C15—C10—C11—C6	−176.9 (6)
C2—Hg1—O1—C17	−152.7 (5)	C6—C11—C12—C13	175.7 (7)
C1—Hg1—O1—C17	25.3 (5)	C10—C11—C12—C13	0.8 (11)
C3—N1—C1—N2	−0.3 (7)	C11—C12—C13—C14	−0.3 (14)
C5—N1—C1—N2	169.5 (6)	C12—C13—C14—C15	0.9 (15)
C3—N1—C1—Hg1	−172.4 (5)	C13—C14—C15—C10	−1.9 (15)
C5—N1—C1—Hg1	−2.5 (10)	C9—C10—C15—C14	−176.3 (8)
C4—N2—C1—N1	0.5 (7)	C11—C10—C15—C14	2.3 (13)
C16—N2—C1—N1	179.3 (6)	C1—N2—C16—C17	73.3 (9)
C4—N2—C1—Hg1	173.4 (5)	C4—N2—C16—C17	−108.0 (8)
C16—N2—C1—Hg1	−7.7 (9)	C18—O1—C17—C16	−166.3 (6)
C2—Hg1—C1—N1	151.3 (9)	Hg1—O1—C17—C16	14.7 (8)
O1—Hg1—C1—N1	142.7 (6)	N2—C16—C17—O1	−66.7 (8)
C2—Hg1—C1—N2	−19.8 (13)	C17—O1—C18—C19	−165.6 (6)
O1—Hg1—C1—N2	−28.3 (5)	Hg1—O1—C18—C19	13.4 (8)
C21—N4—C2—N3	−2.4 (8)	C2—N3—C19—C18	74.7 (10)
C22—N4—C2—N3	177.6 (6)	C20—N3—C19—C18	−104.0 (8)
C21—N4—C2—Hg1	−169.6 (5)	O1—C18—C19—N3	−67.1 (8)
C22—N4—C2—Hg1	10.3 (10)	C2—N3—C20—C21	−0.5 (9)
C20—N3—C2—N4	1.8 (8)	C19—N3—C20—C21	178.4 (7)
C19—N3—C2—N4	−177.1 (7)	N3—C20—C21—N4	−0.9 (9)
C20—N3—C2—Hg1	170.9 (5)	C2—N4—C21—C20	2.1 (9)
C19—N3—C2—Hg1	−8.0 (9)	C22—N4—C21—C20	−177.9 (7)
C1—Hg1—C2—N4	127.6 (10)	C2—N4—C22—C23	99.1 (7)
O1—Hg1—C2—N4	136.1 (7)	C21—N4—C22—C23	−81.0 (8)
C1—Hg1—C2—N3	−38.3 (13)	N4—C22—C23—C24	3.1 (9)
O1—Hg1—C2—N3	−29.7 (5)	N4—C22—C23—C28	−174.7 (5)
C1—N1—C3—C4	0.1 (8)	C28—C23—C24—C25	0.4 (10)
C5—N1—C3—C4	−170.3 (6)	C22—C23—C24—C25	−177.4 (6)
N1—C3—C4—N2	0.2 (9)	C23—C24—C25—C26	1.0 (12)
C1—N2—C4—C3	−0.4 (9)	C24—C25—C26—C27	−0.6 (13)
C16—N2—C4—C3	−179.3 (7)	C25—C26—C27—C32	176.6 (8)
C1—N1—C5—C6	33.7 (10)	C25—C26—C27—C28	−1.3 (12)
C3—N1—C5—C6	−157.7 (6)	C26—C27—C28—C29	−179.7 (7)
N1—C5—C6—C7	−103.0 (7)	C32—C27—C28—C29	2.3 (10)
N1—C5—C6—C11	80.6 (8)	C26—C27—C28—C23	2.6 (10)
C11—C6—C7—C8	0.1 (10)	C32—C27—C28—C23	−175.3 (7)
C5—C6—C7—C8	−176.2 (7)	C24—C23—C28—C29	−179.7 (7)
C6—C7—C8—C9	0.6 (12)	C22—C23—C28—C29	−1.9 (10)
C7—C8—C9—C10	−0.2 (12)	C24—C23—C28—C27	−2.2 (10)
C8—C9—C10—C15	177.6 (8)	C22—C23—C28—C27	175.7 (6)
C8—C9—C10—C11	−1.0 (11)	C27—C28—C29—C30	−2.9 (11)
C7—C6—C11—C12	−176.3 (7)	C23—C28—C29—C30	174.6 (7)
C5—C6—C11—C12	0.0 (10)	C28—C29—C30—C31	0.8 (13)
C7—C6—C11—C10	−1.3 (10)	C29—C30—C31—C32	2.0 (15)
C5—C6—C11—C10	175.0 (6)	C30—C31—C32—C27	−2.6 (14)
C9—C10—C11—C12	176.9 (7)	C26—C27—C32—C31	−177.6 (8)
C15—C10—C11—C12	−1.7 (10)	C28—C27—C32—C31	0.4 (12)

### Hydrogen-bond geometry (Å, °)

**Table:**

*D*—H···*A*	*D*—H	H···*A*	*D*···*A*	*D*—H···*A*
C5—H5A···F3	0.97	2.50	3.384 (9)	151
C5—H5B···F7	0.97	2.39	3.307 (9)	157
C18—H18A···F4^i^	0.97	2.53	3.150 (10)	122
C22—H22B···F9^ii^	0.97	2.46	3.195 (11)	132
C24—H24···N4	0.93	2.50	2.847 (9)	103

Symmetry codes:  (i) −*x*+1, −*y*, −*z*; (ii) *x*+1, *y*−1, *z*.
